# Clinical and Epidemiological Patterns of Scrub Typhus, an Emerging Disease in Bhutan

**DOI:** 10.3390/tropicalmed4020056

**Published:** 2019-03-29

**Authors:** Kezang Dorji, Yoenten Phuentshok, Tandin Zangpo, Sithar Dorjee, Chencho Dorjee, Peter Jolly, Roger Morris, Nelly Marquetoux, Joanna McKenzie

**Affiliations:** 1 School of Veterinary Science, Massey University, Palmerston North 4442, New Zealand; kezangt.dorjee@gmail.com (K.D.); vetyoen@gmail.com (Y.P.); zheynuapa@gmail.com (T.Z.); P.D.Jolly@massey.ac.nz (P.J.); J.S.McKenzie@massey.ac.nz (J.M.); 2 Samdrup Jongkhar Hospital, Ministry of Health, Samdrup Jongkhar 41001, Bhutan; 3 National Centre for Animal Health, Department of Livestock, Ministry of Agriculture and Forests, Serbithang, Thimphu 11001, Bhutan; 4 Dechencholing BHU-I, Ministry of Health, Thimphu 11001, Bhutan; 5 Faculty of Nursing and Public Health, Khesar Gyalpo University of Medical Sciences of Bhutan, Thimphu 11001, Bhutan; s.dorjee@yahoo.co.nz (S.D.); director@rihs.edu.bt (C.D.); 6 Morvet Ltd., Consultancy Services in Health Risk Management and Food Safety Policy and Programs, Masterton 5885, New Zealand; roger.morris@morvet.co.nz

**Keywords:** scrub typhus, One Health, incidence, clinical pattern, descriptive epidemiology, vector-borne disease, emerging disease

## Abstract

Scrub typhus (ST) is a vector-borne rickettsial infection causing acute febrile illness. The re-emergence of ST in the Asia-Pacific region represents a serious public health threat. ST was first detected in Bhutan in 2008. However, the disease is likely to be under-diagnosed and under-reported, and the true impact is difficult to estimate. At the end of 2014, the SD Bioline Tsutsugamushi Test^TM^ rapid diagnostic test (RDT) kits became available in all hospitals to assist clinicians in diagnosing ST. We conducted a retrospective descriptive study, reviewing records from all hospitals of Bhutan to identify all RDT-positive clinical cases of ST in Bhutan in 2015. The aim was to evaluate the burden of ST in Bhutan, describe the demographic, spatial and temporal patterns of disease, and identify the typical clinical presentations. The annual incidence of RDT-positive cases of ST reporting to Bhutanese hospitals in 2015 was estimated to be 62 per 100,000 population at risk. The incidence of disease was highest in the southern districts with a subtropical climate and a high level of agricultural production. The highest proportion of cases (87%) was rural residents, with farmers being the main occupational category. The disease was strongly seasonal, with 97% of cases occurring between June and November, coinciding with the monsoon and agricultural production seasons. Common ST symptoms were not specific, and an eschar was noted by clinicians in only 7.4% of cases, which is likely to contribute to an under-diagnosis of ST. ST represents an important and neglected burden, especially in rural communities in Bhutan. The outcomes of this study will inform public health measures such as timely-awareness programmes for clinicians and the public in high-risk areas, to improve the diagnosis, treatment and clinical outcomes of this disease.

## 1. Introduction

Scrub typhus (ST) or tsutsugamushi disease is a vector-borne rickettsial disease that is caused by the obligate intracellular bacterium *Orientia tsutsugamushi*. The primary reservoir is a trombiculid mite of the genus *Leptotrombidium*, which maintains the infection within populations through both transovarial and transtadial means of transmission [1]. Transmission to humans and other mammals occurs when the larval stage of infected mites feed on a human host [1]. A high risk of exposure to ST is associated with outdoor activities, agricultural work in particular, or living near grasslands or fields [2,3]. Humans are dead-end hosts, with no evidence of horizontal transmission of *O. tsutsugamushi* between people.

The geographic distribution of endemic ST is associated with the distribution of the reservoir mite in an area known as the ‘tsutsugamushi triangle’, centered on South-East and Pacific Asia [4]. ST has been described in this region for over a century [1,5]. The recent resurgence and re-emergence of ST in the endemic area has been associated with global climate change, influencing the distribution of infected mites [1]. Other putative factors are changes in agricultural practices and human behaviour, as well as improvements in diagnostic capabilities [5,6].

ST clinically presents as an acute non-specific febrile illness, which is difficult to diagnose [7]. Other common symptoms include nausea, vomiting, headache, myalgia and respiratory signs [8,9]. An eschar at the site of the bite, occurring before the onset of other symptoms, is pathognomonic [8,10]. However, the presence of an eschar, usually on the front of the body [11], is variably reported in 1–97% of ST patients, depending on the region of the world [1]. While ST is readily treated with antibiotics such as tetracycline, doxycycline, azithromycin and rifampicin [12], the nonspecific flu-like symptoms lead to the under-diagnosis and the under-treatment of this disease in many countries. Untreated ST can cause major complications and ultimately death. The median case fatality rate in untreated patients was estimated to be between 6 and 10% [1,13], but fatalities of up to 70% have been reported [1]. The duration of illness before effective antibiotic treatment is positively associated with progression to severe disease [14]. Hospital-based studies in South India reported a case fatality of 8–9% in ST patients reporting to the hospital, with multi-organ dysfunction observed in 34% of these patients [9,15]. Prompt clinical diagnosis and timely appropriate treatment are thus critical for improving the clinical outcome in individual patients [8,15], and for decreasing the public health impact of this disease [1].

In Bhutan, located within the tsutsugamushi triangle, ST was first identified as a cluster of pyrexia cases of unknown origin reporting to the Gedu hospital in the summer of 2008 [16]. A second outbreak occurred in Gedu in July 2009, of which 70% of cases were confirmed as ST by the Armed Forces Research Institute of Medical Sciences in Bangkok [17]. The disease was made notifiable in Bhutan in 2010. Notifications began to increase, particularly from the southern subtropical regions, which remain hot and humid for most of the year, and are exposed to the Indian summer monsoon [18]. However, given the non-specific presentation of ST, the disease was likely to be under-reported with only 22 to 67 cases being notified annually between 2012 and 2014. Misdiagnosis by clinicians and a lack of awareness amongst the public will respectively result in inappropriate case management, and delays in seeking treatment for febrile illness, in turn increasing the impact of ST in Bhutan.

In 2014, the Ministry of Health of Bhutan initiated a national sero-surveillance programme to gather more information on the incidence of ST, and to raise awareness among clinicians. In Bhutan, 19 of the 20 districts have a government district hospital, in which doctors provide in-patient and out-patient clinical services for the general public. The Gasa district in the far-north Himalayan area only has a Basic Healthcare Unit—Grade 1 (BHU-I) which functions as a district hospital. The country has two regional referral hospitals: in Gelephu, servicing southern Bhutan, and in Monggar, servicing eastern Bhutan. A national referral hospital is located in the capital city, Thimphu. There are no private medical services in Bhutan. As part of the sero-surveillance programme, the Ministry of Health approved the use of a commercial point-of-care rapid diagnostic test (RDT), the SD Bioline Tsutsugamushi Test^TM^, in all hospitals and the BHU-I in Gasa, to support clinicians with the differential diagnosis and timely treatment of ST. The test has the advantage of low cost, rapidity, a single test result and simple interpretation [19]. The SD Bioline Tsutsugamushi Test^TM^ is an immunochromatographic test that is designed for use in clinical settings. It detects IgM, IgG and IgA antibodies against *O. tsutsugamushi*, which increases the sensitivity of the test in patients that may seek treatment once past the acute phase, and those which have been re-infected with *O tsutsugamushi* and have an elevated IgG titre [20].

Under the national sero-surveillance programme, clinicians were encouraged to send samples from patients positive to the RDT to the Royal Centre for Disease Control (RCDC), which functions as the national public health laboratory in Thimphu, for confirmatory testing using the Scrub Typhus Detect TM IgM Enzyme-Linked Immunosorbent Assay (ELISA) test (Inbios International, Inc., Seattle, WA, USA). Cut-off values for the ELISA were calculated for samples collected from Bhutan with the assistance of a laboratory in Pune, India.

Before 2015, no population-wide information on ST was available in Bhutan. With new diagnostic capabilities becoming widely available in hospitals in 2015, a rise in diagnosed ST in Bhutan was expected, irrespective of the level of notification to the Ministry of Health. The aim of this descriptive epidemiology study was therefore to compile the first year of data that was generated by the improved diagnostic capability in hospitals and the national sero-surveillance programme, to gain insight into the epidemiological patterns of ST and the likely impact of ST in Bhutan.

The objectives of our study were to: (1) obtain a more accurate estimate of the incidence of clinical ST, (2) describe the clinical patterns of ST, and (3) describe the demographic, spatial and temporal epidemiological characteristics of clinical ST in Bhutan. The outcomes of this study would contribute to more accurate estimates of the national burden of disease, and support the development and implementation of public health measures to reduce the impact of ST.

## 2. Materials and Methods

We conducted a national descriptive study of laboratory-confirmed ST cases that were identified in hospitals in Bhutan in 2015. The case definition was clinically suspected ST cases that were confirmed by the Rapid Diagnostic Test (RDT; SD Bioline Tsutsugamushi Test^TM^) during routine practice in hospitals in Bhutan during 2015.

### 2.1. Data Collection

ST cases were identified retrospectively by examining the clinical records of all district and referral hospitals and the BHU-I in Gasa district in early 2016. RDT-positive cases that were identified during a hospital-based case control study conducted from October to December 2015 in 11 districts with a higher incidence of ST were included. RDT-positive samples from this case-control study were sent to the RCDC for confirmatory IgM ELISA testing.

Data were obtained from RCDC on IgM ELISA-positive cases tested under the national sero-surveillance programme and the case control study.

For cases that were eligible for the case control study, demographic, clinical and epidemiological information were collected by interview during the study. For other patients, the clinical data were retrieved from the hospital records, and the demographic data was retrieved directly from the patient using their recorded contact details, where necessary.

The national census data issued by the Ministry of Health, Bhutan’s Statistical Bureau, was used as the denominator to calculate national and district-level incidence, and age-based incidence rates of RDT-positive ST cases. The district population, as well as the rural versus urban population data was derived from 2015 statistics and age demographic data from 2017 statistics.

### 2.2. Analysis

We used descriptive methods to analyse the clinical and epidemiological characteristics of ST cases, including demographic, temporal and spatial distributions. Temporal patterns were based on the date of consultation in local hospitals. Spatial distribution was based on the address of the patient at the time of consultation. We mapped the district-level incidence of the RDT+ ST cases with and without the cases identified during the case control study, to identify a potential confounding of the case control study on the spatial distribution of ST. The age distribution between male and female patients was compared by using a two-sample Kolmogorov-Smirnov (KS) test. The proportion of patients experiencing each group of symptoms was compared between males and females using the Pearson’s Chi-squared test. The proportion of rural versus urban cases was compared to that in the general population, using a test of equal proportions between samples.

Incidence rates were expressed as the cumulative number of ST cases per 100,000 persons at risk in 2015.

All analyses were performed using R [21].

### 2.3. Ethical Considerations

The ethical clearance was approved by the Research Ethics Board of Health (REBH), Ministry of Health, Royal Government of Bhutan, Thimphu via letter number REBH/PO/2015/042 on the 25 November 2015.

## 3. Results

A total of 470 RDT-positive clinical cases of ST was identified in this study, representing an observed annual incidence of 62 cases per 100,000 persons at risk in Bhutan in 2015. Among the 470 cases, 160 samples were sent to the RCDC for IgM ELISA testing, as part of the national sero-surveillance programme, of which 85 (53%) were ELISA-positive. A further subset of the 470 cases included 125 RDT-positive samples identified through the case control study which were tested with the IgM ELISA at RCDC, of which 79 (63%) were ELISA-positive.

**Figure 1:**
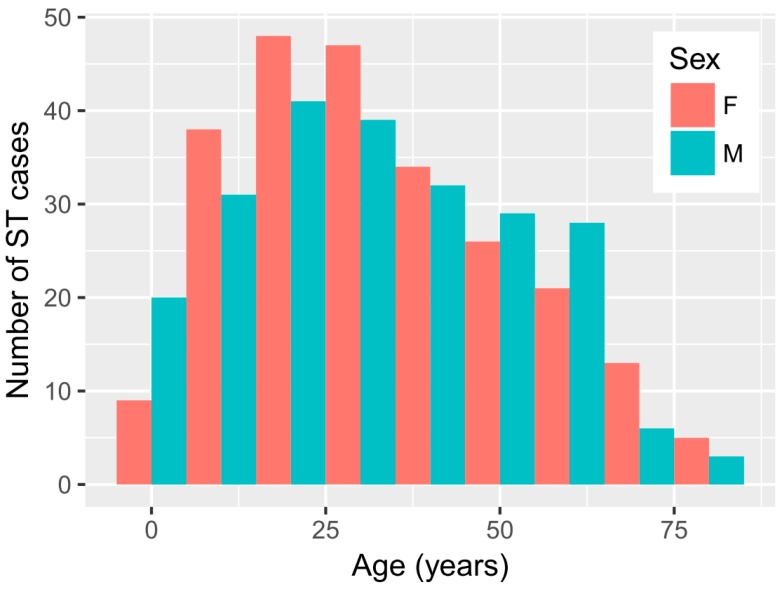
Age distribution of 470 clinically suspected scrub typhus (ST) cases that were positive to the SD Bioline Tsutsugamushi test in Bhutan in 2015.

**Figure 2:**
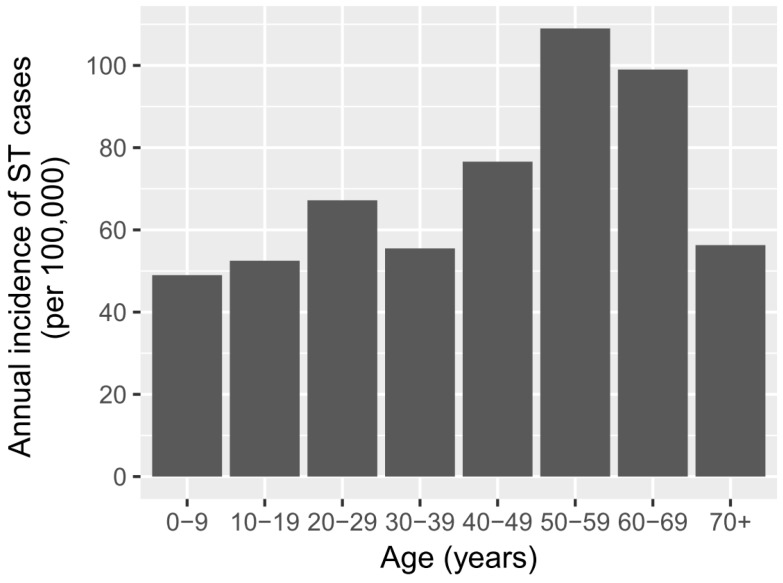
Age-specific incidences of 470 clinically suspected ST cases that were positive to the SD Bioline Tsutsugamushi test in Bhutan in 2015.

There was a similar proportion of females (51.3%) and males (48.7%) among the cases. The median age of ST patients was 30 years old, with the highest percentage of cases within the 20–40-year-old period (Figure 1). The age distribution of ST in males and females was very similar (Figure 1) and statistically not different (KS *p*-value = 0.65). While the highest number of ST cases occurred in the 20–40-year old age group, the age-specific incidence was highest in age groups between 40 and 70 years old (Figure 2).

**Figure 3:**
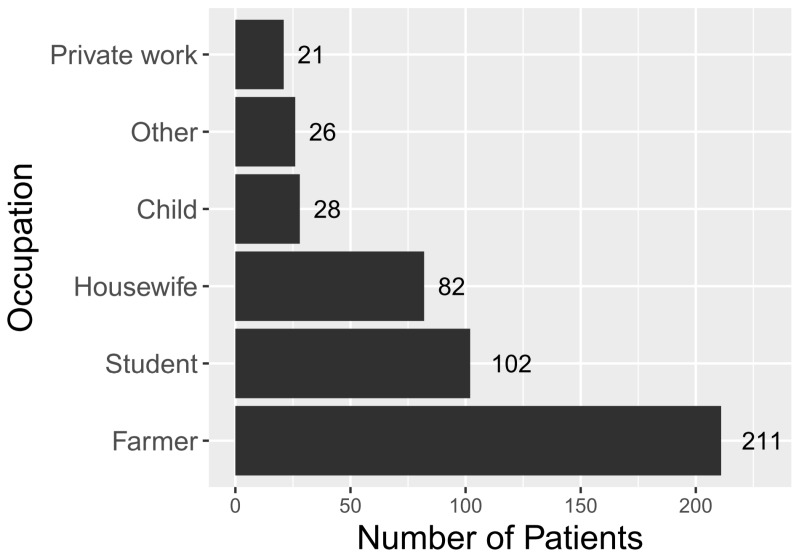
Occupation of 470 clinically suspected ST cases that were positive to the SD Bioline Tsutsugamushi test in Bhutan in 2015 (the “Other” category encompasses gomchen (lay priests), patients from India, military personnel, monks and civil servants).

Farmers represented the main line of occupation among ST patients (45%), followed by students (22%) and housewives (17%) (Figure 3). The vast majority of patients were from rural areas (88%, *n* = 412) compared to 62% in the general population. Rural cases were overrepresented in the RDT-positive population, compared to the general population (*p* < 0.0001).

**Figure 4:**
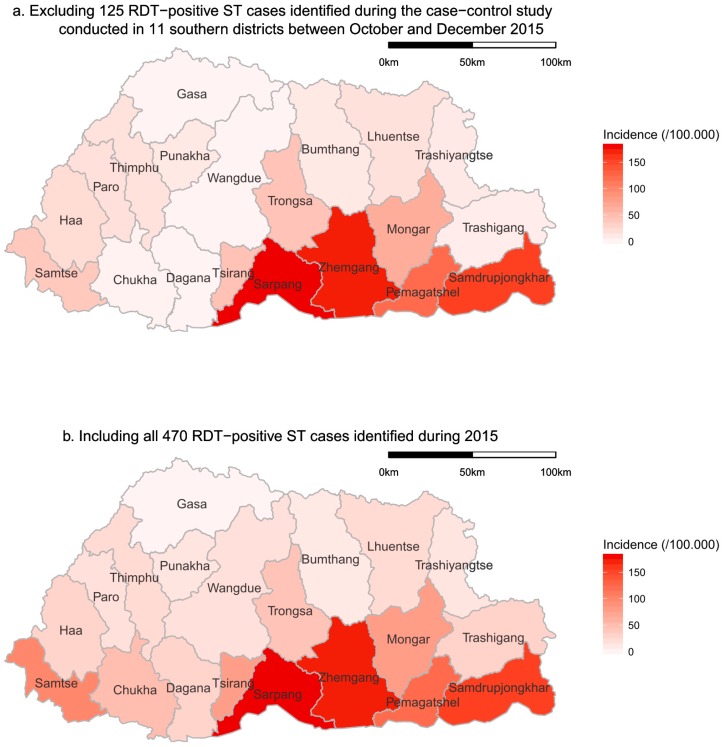
Spatial distribution of the incidences of clinically suspected scrub typhus cases that tested positive to the SD Bioline Tsutsugamushi test in Bhutan in 2015 ((**a**) excluding 125 RDT-positive ST cases that were identified during the case-control study conducted in 11 southern districts between October and December 2015, (**b**) including all 470 RDT-positive ST cases that were identified during 2015).

The highest annual incidence of RDT-positive ST cases was observed in sub-tropical districts in the south of Bhutan (Figure 4). This spatial pattern was consistent with and without the cases identified through the case control study, which was conducted in the southern districts (Figure 4). Cases were observed in all districts except Gasa, which is located in the higher Himalayas.

**Figure 5:**
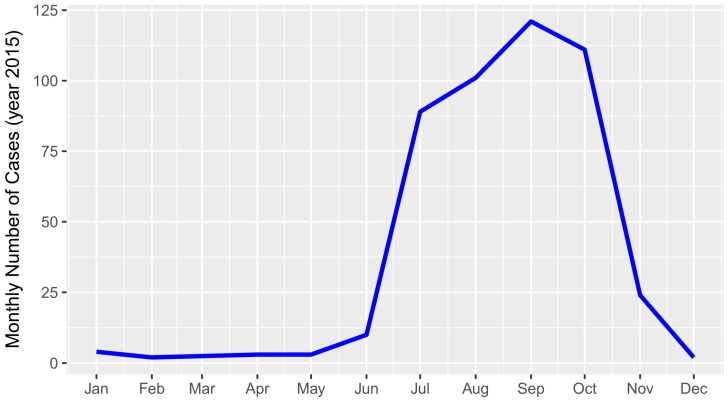
Temporal distribution of 470 clinically suspected scrub typhus cases that tested positive to the SD Bioline Tsutsugamushi test in Bhutan in 2015.

The temporal distribution of ST cases showed a strong trend of seasonality that was associated with the monsoon season. There were virtually no cases between December and June, followed by a sharp increase in July with a peak of incidence in September, and a sudden drop from October onwards (Figure 5).

Most RDT-positive ST cases presented with nonspecific flu-like symptoms. Fever was present in all patients, since this was generally the criterion for clinicians to perform the RDT, headache in 87%, myalgia and poly-arthralgia, respectively, in 56% and 40% of patients, and diarrhea or vomiting in 22% (Table 1). The presence of a pathognomonic eschar was only observed in 7.4% of the patients, twice as commonly in males (10%) as females (5%) (*p* = 0.06). Males and females mostly presented with a similar pattern of symptoms. However, respiratory and vascular symptoms were significantly 1.7 (*p* = 0.01) and 1.9 (*p* = 0.04) times more frequent in males.

## 4. Discussion

The emergence of reported ST cases in Bhutan between 2010 and 2014 indicated the potential for ST to be a significant health issue in Bhutan. However, most healthcare workers as well as the public were not aware of the disease at that time. This is the first national study of ST in Bhutan, providing more accurate evidence for the overall incidence and the spatial, temporal and demographic distributions of ST in Bhutan’s population in 2015.

The identification of 470 cases of RDT-positive ST in Bhutan in 2015, representing an observed annual incidence of 62 RDT-confirmed clinical ST cases/100,000 persons at risk, is a much higher incidence of ST than that reflected through the national sero-surveillance data from previous years. These results suggest that ST is a common cause of febrile illness in Bhutan. However, there are many challenges that are associated with serological testing for ST that make it difficult to interpret the extent to which this result reflects the true incidence of clinical ST in the Bhutanese population. Given the SD Bioline Tsutsugamushi test measures IgM, IgG and IgA antibodies it is likely to overestimate the incidence of clinical ST infections that occurred during 2015, given the test also captures antibodies that are associated with historical exposure in an unknown proportion of febrile cases [20]. This is supported by the IgM ELISA test results being positive in only 53% and 63% of the subset of RDT+ cases that were tested with the ELISA, respectively, in the sero-surveillance programme and the case control study. The IgM test results are likely to reflect the proportions of RDT+ cases that have current infections. The InBios Scrub Typhus Detect IgM ELISA was reported to have a sensitivity and specificity of 93% and 91% respectively, when measured against patients with typhus-like illness that fulfilled robust scrub typhus criteria in Thailand [22]. On the other hand, reinfection with *O. tsutsugamushi* is not uncommon in endemic areas, and such cases can be expected to have a rising IgG titre, which would not be captured in the IgM ELISA test results [20]. Furthermore, a proportion of cases who sought treatment after only a few days of fever may not have developed IgM antibodies, and this would have been missed by both serological tests [20]. In general, the RDT-positive incidence is more likely to reflect the overall exposure to *O. tsutsugamushi* amongst febrile patients, including recent and historical exposure, while the IgM ELISA incidence is more likely to reflect clinical disease that is associated with recent exposure. Both tests are likely to underestimate the true incidence of clinical ST in the study populations. Due to ST’s non-specific flu-like symptoms, it is likely that not all cases would have sought treatment at the hospitals; milder or self-limiting cases of ST might not be seen in the hospital. The disease is also likely to be under-diagnosed, due to a lack of awareness of clinicians and non-specific clinical presentation. Moreover, anecdotal reports indicate that clinicians may have faced a shortage in the supply of RDT kits in some district hospitals in 2015, especially in high-incidence areas, and an unknown number of clinical ST cases may not have been tested.

There is considerable variation in the information on sensitivity and specificity of the SD Bioline Tsutsugamushi test. While the company selling the test claims a sensitivity and specificity of 99% and 96% respectively, one study evaluating this test against the immunofluorescence assay (IFA) in Thailand found a sensitivity of only 20.9% (95% CI: 10.0–36.0) in acute samples that are collected from patients who had a fever for a mean of 2–3 days. A higher sensitivity of 76.7% (95% CI: 61.4–88.2) was found in convalescent samples that were collected from the same patients at a median interval of 14 days (range 11–30 days). The specificity was similar in acute (74.4%; 95% CI: 58.8–86.5) and convalescent (76.7%; 95% CI: 61.4–88.2) samples [23]. Other studies have found that the same test had a sensitivity of 66.7% (95% CI 57.1–75.1%) and 72.6% in acute samples from febrile patients in Thailand and Korea, respectively, when assessed against the IFA [20,24]. Specificity was estimated to be 98.4% (95% CI: 91.5–99.7) in the Thai study [20]. Furthermore, there are acknowledged problems with evaluating the test accuracy by using the IFA as a gold standard, which may result in underestimates of sensitivity and specificity in the tests being evaluated [22]. The authors recommend the widespread use of the IgM ELISA, with optical density cut-offs, calibrated for an individual country, as a gold standard for the acute diagnosis of ST infections. The accuracy of serological tests may also be influenced by the variation in the circulating strains of *O. tsutsugamushi* [22,24]. The authors acknowledge the need for ST diagnostic tests to be validated within the country in which they are to be used, to provide more accurate results. The SD Bioline Tsutsugamushi test has not been validated for Bhutan, which further complicates the interpretation of how well the results represent the true ST situation in the country.

While there are challenges in interpreting how well the results of the RDT testing of clinically suspected ST cases reflect the true incidence of clinical ST in the national Bhutanese population, the results are reasonably comparable between sub-groups within the tested population, given that the same test was used throughout the country. It was previously thought that ST was endemic only in the southern part of the country. However, this study has shown that while the highest incidence of RDT-positive clinical ST cases occurred in the subtropical southern districts of Bhutan, cases occurred throughout Bhutan in all but one district, Gasa, which is located in the higher Himalaya area (Figure 4). Hence, ST is a vector-borne zoonotic disease of national importance. The higher incidence in subtropical areas is likely to be associated with the climate favouring the survival of chigger mites, together with higher levels of exposure to the vector through the more intensive farming activities undertaken in the favourable growing climate in this region. The lack of cases in the mountainous Gasa district may be due to an unsuitable environment for the reservoir’s sustenance. However, it may also be influenced by the district not having a district-level hospital, which may have led to potential ST cases not being diagnosed and/or tested. Furthermore, the local population may experience difficulty in accessing medical services in this mountainous area, which could further contribute to an underestimation of the incidence of ST in this district.

There was a very strong temporal pattern in the occurrence of RDT-positive clinical ST cases, with 97% of cases observed between June and November, peaking in September (Figure 5). A similar pattern was found in a study in West Bengal, India [25]. This time of the year coincides with the monsoon and the cropping season, which might favour both the density of the chiggers and people’s exposure to the vector through agricultural work.

The observed seasonal and spatial patterns of RDT-positive clinical ST cases could be affected by clinicians in high-risk areas having greater awareness of ST during the high-risk season. Data on the total number of clinically suspected ST cases and the number of cases tested with the RDT in hospitals were not available. Hence, we cannot assess the consistency with which the RDT was applied across the clinically suspected ST cases in all hospitals, nor the variation in the proportion of RDT-tested cases that were positive. However, it is unlikely that clinicians’ prior knowledge of ST biased the results in this study. While clinicians in the southern areas of Bhutan are generally aware of a higher incidence of febrile disease during the monsoon season, they were mostly unaware of ST and its epidemiology at the time of the study, hence variable awareness of ST is unlikely to have had a significant effect on the clinical suspicion of ST, or the application of the RDT.

The case control study on clinical ST cases diagnosed from October to December 2015 in 11 districts in the south of Bhutan is likely to influence the observed spatial and temporal patterns, as clinicians would have had a greater awareness of ST during the study. Given the study did not begin until October 2015, it would not have influenced the increased incidence showing from July until the peak in September. Furthermore, it would provide more confidence that the incidence declined significantly in November and December. Mapping the RDT-positive ST cases without those from the case control study showed the same spatial pattern with a higher incidence in the southern districts of Bhutan. This gives confidence that there is a higher risk of ST in the subtropical southern districts of Bhutan. A possible source of bias in the spatial and temporal distribution of diagnosed ST may have arisen from a shortage of kits in some hospitals. There is no data available on the timing of diagnostic kit supply to district hospitals during 2015. However, anecdotal reports indicate that some hospitals experienced a temporary shortage of diagnostic kits, particularly hospitals in the high-incidence areas during the high-incidence time of year. This might have influenced the observed distribution of diagnosed cases.

ST predominantly affected people living in rural areas (87%), primarily farmers (45%) (Figure 3). This is consistent with previous observations throughout South-East Asia [25,26,27,28]. Students were the second-highest group with clinical ST. This is consistent with results from previous studies, highlighting the importance of ST in the differential diagnosis of children with pyrexia of unknown origin [29].

The highest age-specific incidence of clinical ST occurred in 40–70 year olds. The national statistics [30] show that a larger proportion of people aged 40 years and over live in rural areas (72%) compared with urban areas (28%). Hence, this age-related result is likely to be confounded with a higher risk of exposure of older people to rural environments and agricultural activities. It may also reflect the cumulative exposure of the at-risk population to *O. tsutsugamushi*, which is captured by the RDT measuring IgG and IgA, as well as IgM antibodies. Further studies that include multi-variable analyses would provide more accurate indications of the risk factors for ST.

Cases of ST in Bhutan presented as a nonspecific febrile illness, with signs of myalgia or poly-arthralgia in half the patients, consistent with reports in the literature [13,31]. Respiratory and vascular symptoms were significantly more frequent in males compared to females (respectively 1.7 and 1.9 times). Such a difference has not been reported in other studies, and it is difficult to propose an explanation for these differences. It may be confounded by variable distribution of these conditions between males and females in the over 40 year old age group. The presence of an eschar at the site of the chigger bite is described as pathognomonic [9], and it can thus be a precious diagnostic clue for clinicians. In Japan, 87% of patients presented with an eschar [27]. In contrast, this lesion was noted by the clinician in only 7.4% of patients (10% in males and 5% in females) in the present study. It is possible that clinicians did not thoroughly investigate patients for this lesion, especially female patients. The low detection rate of eschars may also be influenced by the inclusion of febrile patients with historical exposure to ST in the RDT-positive population, given the test measures of IgG and IgA, as well as IgM antibodies.

Among the 470 RDT-positive cases identified in hospitals, samples from only 160 cases were submitted from only four hospitals to the RCDC under the national sero-surveillance programme. The hospitals were: the national referral hospital in Thimphu, the regional referral hospitals in Sarpang and Mongar, and the secondary referral hospital in Paro. District hospitals in the rest of the country did not contribute samples, due to the perceived difficulty of transporting the samples to Thimphu, and a general lack of awareness about the sero-surveillance programme. As a result, the data generated through the national sero-surveillance programme significantly under-represents the true incidence of clinical ST in Bhutan. Raising clinicians’ awareness of the sero-surveillance programme and ensuring facilities are available to transport samples from district hospitals to the RCDC in Thimphu would improve the representativeness of the sero-surveillance data. This would improve the ability of the Ministry of Health to monitor trends and to determine if clinical ST continues to emerge over time and over geographic areas in Bhutan. It would also indicate hospitals that may be under-diagnosing ST, which would enable targeted measures to be implemented to improve treatment-seeking behaviours of the public, and/or the diagnosis and treatment of ST in such areas where there is a difference between the expected and actual reported cases.

Despite the limitations of the SD Bioline Tsutsugamushi test, such commercial point of care tests are considered to be useful in clinical settings in limited resource environments to guide the differential diagnosis of ST [32], with the advantages of affordability, rapidity, single test results and ease of interpretation [19]. However, the tests need to be used with appropriate clinical discernment. It is important for clinicians to rule out other causes of acute febrile illness and the possibility of co-infection of patients with other infections that are associated with similar exposures, such as dengue and other rickettsial diseases. Given the limitations in the sensitivity of the test, clinicians should be encouraged to initiate treatment in patients in which they strongly suspect ST, even if the sample tests negative to the RDT, and to send samples from such patients to RCDC for confirmatory testing with the IgM ELISA. The epidemiological information generated through this study provides supplementary information that clinicians can use to diagnose ST, even in the absence of a positive RDT. Furthermore, epidemiological information can significantly contribute to the formulation and timely implementation of public health measures to reduce the burden of ST in Bhutan. Communication programmes can be implemented in all areas, to raise the awareness of the public to seek treatment if they are suffering flu-like symptoms. Likewise, providing clinicians with guidelines and professional training in risk factors for ST, such as the times of year, geographic areas, occupations and types of exposure to potential chigger habitat could increase the probability of considering ST in the differential diagnosis for patients presenting with nonspecific febrile illness. The timely supply of test kits and therapeutic drugs to hospitals, especially in high-risk areas, may contribute to the improved diagnosis and treatment of ST cases.

This study represents an important step towards understanding the incidence and associated risk factors for ST in Bhutan, which can inform both public health measures and the design of future studies. It would be valuable to validate the SD Bioline Tsutsugamushi test in Bhutan. Furthermore, studies to identify the antigenic variants of *O. tsutsugamushi* in Bhutan and in neighbouring areas of northern India could help with the development of more sensitive serological tests. Given the complex nature of this vector-borne zoonotic disease, One Health studies, involving collaboration between multiple sectors and disciplines, are required to explore the roles of the environmental and anthropologic factors, and the ecology of the reservoir and vector population, to understand the drivers of the spatial, temporal and demographic distributions of ST in Bhutan.

## Figures and Tables

**Table 1:** Comparison of the clinical manifestations of 470 clinically suspected ST cases that tested positive to the SD Bioline Tsustugamushi test in males (241) and females (229) in Bhutan in 2015.

	Positive	*n*	Proportion (Overall)	Proportion (Males)	Proportion (Females)	*p*-Value
Fever	470	470	100%	100%	100%	NA
Headache	407	470	86.6%	83.8%	89.2%	0.12
Myalgia	265	470	56.4%	55.9%	56.8%	0.91
Malaise	216	468	46.2%	48.7%	43.8%	0.33
Polyarthralgia	189	470	40.2%	41.5%	39%	0.65
Other Digestive Symptoms	179	470	38.1%	37.1%	39%	0.74
Chills	130	469	27.7%	28.1%	27.4%	0.95
Diarrhoea/Vomiting	102	470	21.7%	21%	22.4%	0.79
Giddiness	95	467	20.3%	16.8%	23.7%	0.09
Respiratory Symptoms	91	470	19.4%	24.5%	14.5%	0.01
Rashes	88	469	18.8%	18.8%	18.8%	1
Vascular Symptoms	45	470	9.6%	12.7%	6.6%	0.04
Eschar	35	470	7.4%	10%	5%	0.06
Restlessness	31	470	6.6%	7%	6.2%	0.88
Lymphadenopathy	9	470	1.9%	2.2%	1.7%	0.94
Jaundice	3	470	0.6%	0.4%	0.8%	1
